# Prevalence of premenstrual syndrome in Iran: A systematic review and meta-analysis

**Published:** 2017-11

**Authors:** Mehdi Ranjbaran, Reza Omani Samani, Amir Almasi-Hashiani, Pegah Matourypour, Ashraf Moini

**Affiliations:** 1 *Department of Epidemiology and Reproductive Health, Reproductive Epidemiology Research Center, Royan Institute for Reproductive Biomedicine, ACECR, Tehran, Iran. *; 2 *Department of Medical-Surgical Nursing, Nursing and Midwifery Faculty, Tehran University of Medical Sciences, Tehran, Iran. *; 3 *Department of Endocrinology and Female Infertility, Reproductive Biomedicine Research Center, Royan Institute for Reproductive Biomedicine, ACECR, Tehran, Iran.*; 4 *Department of Gynecology and Obstetrics, Arash women's Hospital, Tehran University of Medical Sciences, Tehran, Iran. *

**Keywords:** Premenstrual syndrome, Iran, Systematic review, Meta-analysis

## Abstract

**Background:**

Premenstrual syndrome (PMS) is a common disorder characterized by physical, mental and behavioral changes in the luteal phase of the menstrual cycle in the reproductive age women.

**Objective:**

The present study aimed to determine the overall prevalence of PMS in Iran by a systematic review and meta-analysis study.

**Materials and Methods:**

In this systematic review and meta-analysis, we searched international databases included ISI Web of Knowledge, PubMed/Medline, Scopus, Google Scholar, and also local databases including Iranmedex, Scientific Information Database, and Magiran for articles in English and Persian language published up to September 2016. We carried out data analysis with Stata version 11. We examined heterogeneity in the results of studies through I^2^ statistics and Chi-square based Q test. Also, we investigated the effects of potential heterogeneity factors in the prevalence of PMS by meta-regression.

**Results:**

We studied a total of 9147 reproductive-age women from 24 articles which entered to meta-analysis. Based on the result of random effect model, we estimated the overall prevalence of PMS 70.8% [95% CI: 63.8-77.7]. The results of subgroup analysis revealed that prevalence of PMS was 80.4% (95% CI; 66.9-93.9) among high school students, 68.9% (95% CI; 59.2-78.6) among university students, and 54.9% (95% CI; 51.6-58.2) in general population. Univariate meta-regression model showed that prevalence of PMS was decreased by increasing the age of subjects but this was not statistically significant (p=0.155).

**Conclusion:**

Our finding showed that PMS was prevalent in Iranian reproductive age women especially among high school students. More epidemiological research for determining factors that affect PMS prevalence seems essential.

## Introduction

Premenstrual syndrome (PMS) is a common disorder characterized by physical, mental and behavioral changes in the luteal phase of the menstrual cycle in the reproductive age women (between menarche and menopause). PMS starts 6-12 days before menses and persists for two days (maximum four days) after the onset of menses (1, 2). Reproductive age in women; years of life between menarche and menopause, has different range among countries in the world but overall it is about 15-49 yr (3, 4). 

Hippocrates was the first one who introduced the association between menstrual periods and behavioral changes (5). In 1931 Robert Frank an American physician at New York Academy of Medicine attributed these changes to ovarian dysfunction and used the term “premenstrual tension”. In the same year, Karen Horney believed that this syndrome can be caused by sexual desire and so-called “premenstrual syndrome” (5, 6).

Clinical symptoms of PMS include physical symptoms such as swelling, breast tenderness, headache, increased appetite, heart palpitations and behavioral and psychological symptoms such as depression, irritability, fatigue, aggression, suicidal tendencies, poor concentration, mood swings, and social withdrawal (5, 7, 8). PMS is the common untreated disorder and a public health problem among reproductive-age women, that, adversely affects mental well-being, quality of life and academic performance (9). Based on a systematic review and meta-analysis reported, the prevalence of PMS is 48% in the world (1). In addition, according to studies reported from Iran, the prevalence of PMS is varies between 30% and 99.5% (10-12).

In Iran, multiple social roles of women are determining their health status and leading to different health-related consequences. Thus, to plan the effective preventive intervention, providing valid and reliable evidence in this field is necessary because of the variability of the available studies results. Although recently reported reviews have provided useful results on other aspects of women health problem, there is no review of various studies result in Iran on the prevalence of PMS (13, 14).

Therefore present study was aimed to determine the overall prevalence of PMS as another health problem in women in Iran using systematic review and meta-analysis.

## Materials and methods

**Search strategy**

In this systematic review and meta-analysis, we searched international databases included ISI Web of Knowledge, PubMed/ Medline, Scopus, Google Scholar and local databases including Iranmedex, Scientific Information Database, and Magiran systematically for both English and Persian language articles that published up to September 2016. We used keywords such as [(“Premenstrual Syndrome” OR “Menstrual disorders”) AND “Iran”].

**Exclusion and inclusion criteria**

We selected studies according to the following criteria: 1) the study published in English or Persian language and conducted in Iran 2) the study presented original data, and 3) the study reported the prevalence of PMS. We excluded studies in special populations such as employed women, studies with invalid tools for PMS, duplicated articles, and studies with design other than cross-sectional studies that not reported the prevalence of PMS.

**Quality assessment**

Two researchers independently assessed full text of eligible articles using strengthening the reporting of observational studies in epidemiology checklist and evaluated the main issues such as sampling method and validity of the measurements (15). In the case of disagreement, two reviewers discussed with a third reviewer to reach consensus.

**Statistical analysis**

We carried out data analysis by Stata version 11 software. We used the binomial distribution to calculate the standard error in each study. We examined the heterogeneity in the results of different studies through Chi-square based Q test with a significant level of p<0.1 and I^2^ statistics with values above 75% as significant heterogeneity (16). We used subgroup analysis to determine the pooled estimated of PMS prevalence based on the study population. Also, we investigated the effects of the potential heterogeneity factors in the prevalence of PMS by Moment base meta-regression model.

## Results

**Figure 1:**
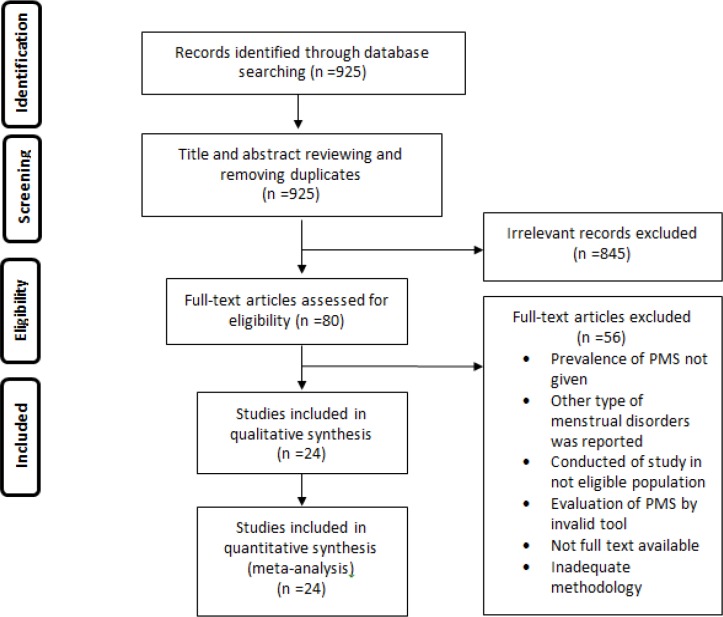
Flowchart for selection of studies about the prevalence of premenstrual syndrome in Iran

Figure 1 displays the flowchart of article selection according to PRISMA flow diagram. In the first step, we identified 925 records through all databases search. After screening and removal of duplicate papers, 80 full-text papers identified for eligibility. Finally, 24 papers interred in this meta-analysis (5, 6, 8, 10, 11, 17-35). 

Table I shows the characteristics of 24 selected studies for the systematic review and meta-analysis. Total sample size in this 24 studies was 9147 reproductive age women. 

**Figure 2:**
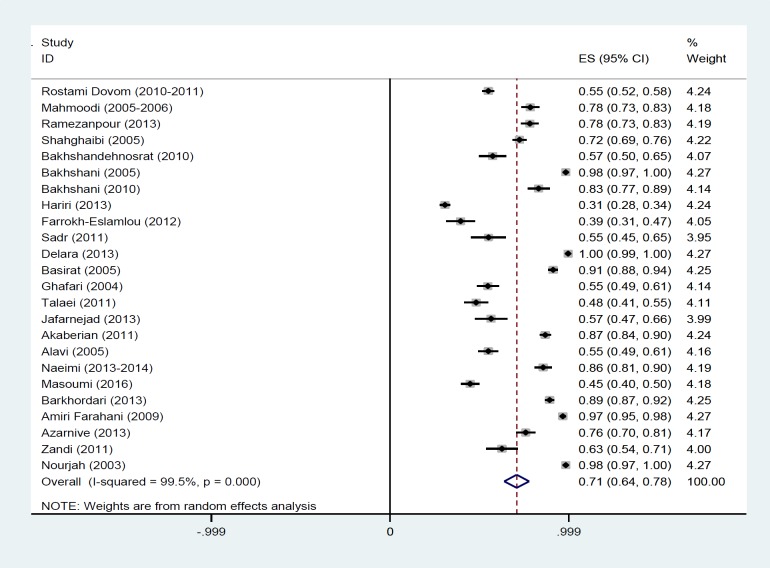
Forest plot of studies about the prevalence of premenstrual syndrome in Iran

The results of Chi-square test based on Q test and I^2^ statistics revealed high heterogeneity between the results of the studies (Q=4657.44, p<0.001, and I^2^=99.5%) and consequently, we used random effect model for the meta-analysis. As presented in figure 2, based on the result of this model we estimated the overall prevalence of PMS 70.8% (95% CI: 63.8-77.7). The size of each Rectangle represents the weight of each study and the lines around it were 95% confidence interval. Diamonds and the vertical dashed line represent the overall estimated prevalence.

**Figure 3.**
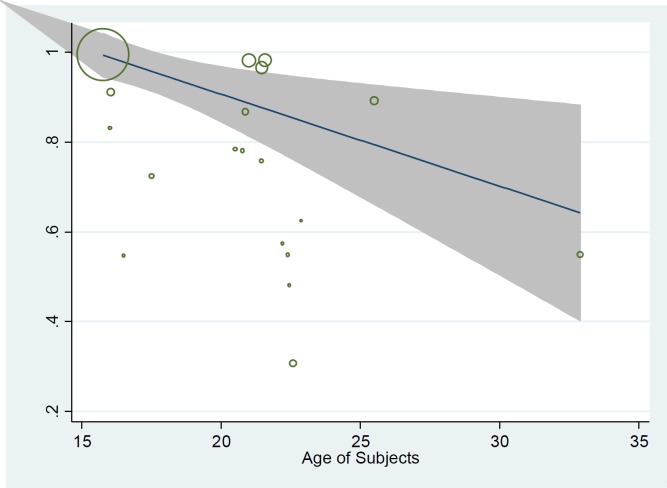
Meta-regression graph of the prevalence of PMS based on the age of subjects

The results of subgroup analysis revealed that prevalence of PMS was 80.4% (95% CI; 66.9-93.9) among high school students, 68.9% (95% CI; 59.2-78.6) among university students and 54.9% (95% CI; 51.6-58.2) among the general population (18-45 yr old). In addition subgroup analysis based on diagnostic criteria showed that prevalence is slightly low in DSM-IV (68.4%, 95% CI; 57.1-79.7) compared to other methods (72.1%, 95% CI; 62.2- 82.0). We used meta-regression model to determine the effect of some potential variable in heterogeneity between studies including the age of participant, sample size and year of study (Table II). The univariate model showed that prevalence of PMS was decreased by increasing the age of subjects but it was not statistically significant (Table II, Figure 3). The situation was similar for the year of study. Moreover, we didn’t find any association between sample size (p=0.459) and diagnostic criteria of studies (p=0.673) with the reported prevalence. Finally, we entered variables with p<0.2 into multiple model and results showed that none of them was significant (p>0.05).

**Table I:** Characteristics of included studies in meta-analysis

**References**	**Location**	**Subjects**	**Diagnostic** **criteria**	**Mean age of participants (±SD)**	**Number**	**PMS**
Rostami dovom (17)	4 Provinces*	General population	ACOG	32.9 ± 7.6	892	54.9
Mahmoodi (20)	Tehran	University students	APA	18-23	255	78.43
Ramezanpour (5)	Gonabad	University students	DRSP	20.77 ± 2.49	270	78.1
Shahghaibi (19)	Sanandaj	High school students	RMQ gynecologist opinion	17-18	511	72.4
Bakhshandehnosrat (18)	Golsetan	Medical students	ACOG	22.2 ± 2.5	162	57.4
Bakhshani (31)	Zahedan	University students	RMQ	21.64 ± 2.13	300	98.2
Bakhshani (24)	Zahedan	High school students	ACOG	14-18	142	83.1
Hariri (29)	Tehran	University students	PSST	22.58 ± 3.16	925	30.7
Farrokh-eslamlou (30)	Urmia	Medical students	DSM-IV	-	142	39.4
Sadr (28)	Tehran	Medical students	DSM-IV	-	100	55
Delara (11)	Sabzevar	High school students	PAS	15.76 ± 1.1	1379	99.5
Basirat (21)	Babol	High school students	RMQ	16.03 ± 1.15	410	91.18
Ghafari (23)	Ramsar	High school students	RMQ	16.5 ± 1.11	252	54.7
Talaei (22)	Mashhad	Medical students	DSM-IV	22.45 ± 2.35	210	48.1
Jafarnejad (8)	Mashhad	Medical students	DSM-IV	-	115	56.52
Akaberian (10)	Boushehr	University students	DSM-IV	20.88 ± 2.4	478	86.8
Alavi (25)	Bandar Abbas	Medical students	ICD-10	22.4	302	54.9
Naeimi (6)	Sistan and Baluchestan	University students	ACOG and DSM-IV	-	201	85.6
Masoumi (32)	Hamedan	Medical students	PMS symptoms questionnaire	-	356	44.7
Barkhordari (33)	Tehran	Medical students	DSM-IV	17-34	571	89.2
Amiri farahani (26)	Arak	Medical students	Hallbridge *et al.* questionnaire	21.47 ± 2.55	500	96.6
Azarnive (27)	Zabol	University students	DSM-IV	21.45 ± 2.34	240	75.8
Zandi (34)	Parand-Tehran	University students	DSM-IV	22.87 ± 3.64	114	62.5
Nourjah (35)	Tehran	University students	DSM-IV	18-24	320	98.2

ACOG: American college of obstetricians and gynecologists

APA: American psychiatric association

DRSP: Daily record of severity of problems chart

RMQ: Researcher made questionnaire

PSST: Premenstrual symptoms screening tool

PAS: Premenstrual assessment scale

PMS: Premenstrual syndrome

*: Qazvin, Golestan, Kermanshah and Hormozgan

**Table II:** Meta regression analysis of suspected variables of heterogeneity in prevalence of premenstrual syndrome in Iran

**Variable**	**Univariate model**			**Adjusted model** a		
	**Coefficient**	**SE**	**p-value**	**Coefficient**	**SE**	**p-value**
Mean age of participants	-0.0171	0.0115	0.155	-0.0160	0.0122	0.209
Sample size	0.0001	0.0001	0.459	-	-	-
Year of study	-0.0164	0.0120	0.186	-0.0044	0.0134	0.720
Diagnostic criteria^b^	-0.038	0.0889	0.673	-	-	-

a : For variables with p ≤0.2 in the univariate model (Mean age of participants and year of study)

b : DSM-IV vs. other methods

## Discussion

In this meta-analysis, we estimated the pooled prevalence of PMS in Iran and it was 70.8% (95% CI: 63.8-77.7). Based on the results of a meta-analysis of 17 studies recently reported by Direkvand-Moghadam and colleagues the overall prevalence of PMS in the world is 47.8% (1). They included only one study from Iran with a PMS prevalence of 98% (31) and reported that the highest prevalence among all studies has been seen in Iran. But the result of our study showed that, although the prevalence is higher in Iran than the world estimate, it does not reach 98% which reported in their study. 

The prevalence of PMS was reported from Taiwan is 39.85% in female university students (36), and 12.2% in French which conducted among a nationally representative sample of women in reproductive age (37). Similarly, a study reported from India show 18.4% prevalence of PMS among college students (38). The results of our study were somewhat in agreement with the results of some studies that reported high prevalence of PMS such as; study in adolescent girls in Turkey with the prevalence of 61.4% (39), in Japanese high school students with the prevalence of 64.6% (40), in Spanish fertile age women with prevalence of 73.7% (41), and in female undergraduate students of the University of Calabar, Nigeria with the prevalence of 85.5% (42).

These differences between the studies are most probably due to the difference in their study population, sample size, design, and methods of PMS measurement (1, 37). Tolossa and Bekele believe that health sciences students have academic stress which causes high prevalence of PMS (43). Potter *et al.* believe that low prevalence in their study may be because of retrospective design of study and methods of data collection (37). 

Direkvand-Moghadam and colleagues found that increase in sample size is related to a reduced prevalence of PMS. Also, they believe that factors such as physical activity and nutrition can be the source of heterogeneity in the prevalence of PMS among studies (1). In the study by Bianco *et al.* lifestyle, nutrition, and general health were associated with PMS (44).

In a study conducted among female students of Mekelle University College of Health Sciences, in northern Ethiopia in 2013, 83.2% of participants have had at least one PMS symptoms with their menstrual period but according to DSM-IV, the prevalence was 37.0% (43). Results of subgroup analysis based on diagnostic criteria in our study showed that, the prevalence was slightly low in DSM-IV method supporting this finding.

Age of participant might be other justification for differences in the results of studies because PMS is higher among the younger age group (45). As previously reported, the PMS prevalence in the French study among a nationally representative sample of women was low. In addition, the results of subgroup analysis in our study showed high prevalence among high school students who have a lower age, compare to college students of the general population and in meta-regression analysis; PMS was decreased by increasing the age of participant with a gentle slope. High stress in adolescents due to physiological changes, educational pressure and sexual health can lead to increased prevalence of PMS (46).

The main limitation of this systematic review and meta-analysis was the differences in diagnostic criteria of PMS between included studies, and due to the small number of studies in each criteria, we couldn’t perform subgroup analysis based oneach criteria and we only compared DSM-IV method with other criteria.

## Conclusion

Our finding showed that PMS was prevalent in Iranian reproductive age women especially among high school students. More epidemiological research for determining factors associated with PMS seems essential.

## References

[1] Direkvand-Moghadam A, Sayehmiri K, Delpisheh A, Sattar K (2014). Epidemiology of Premenstrual Syndrome (PMS)-A systematic review and meta-analysis study. J Clin Diagn Res.

[2] Kleinstäuber M, Schmelzer K, Ditzen B, Andersson G, Hiller W, Weise C (2016). Psychosocial Profile of Women with Premenstrual Syndrome and Healthy Controls: A Comparative Study. Int J Behav Med.

[3] Golshiri P, Akbari M, Abdollahzadeh MR (2016). Age at Natural Menopause and Related Factors in Isfahan, Iran. J Menopausal Med.

[4] Bahrami N, Soleimani MA, Chan YH, Ghojazadeh M, Mirmiran P (2014). Menarche age in Iran: A meta-analysis. Iran J Nurs Midwif Res.

[5] Ramezanpour F, Bahri N, Bagheri L, Fathi Najafi T (2015). Incidence and Severity of Premenstrual Syndrome and its relationship with Social and Demographic Characteristics among Students’ College, Gonabad-2013. Iran J Obstet Gynecol Infertil.

[6] Naeimi N (2015). The Prevalence and Symptoms of Premenstrual Syndrome under Examination. J Biosci Med.

[7] Deshpande A, Mehvish M (2016). Effect of premenstrual syndrome on cardiovascular parameters and body weight in first-year medical students. J Evol Res Hum Physiol.

[8] Jafarnejad F, Shakeri Z, Najaf Najafi M, Salehi Fadardi J (2013). Evaluation the Relationship between Stress and the Risk of Premenstrual Syndrome. Iran J Obstet Gynecol Infertil.

[9] Brohi ZP, Haider G, Zehra N, Amna A (2011). Frequency and Impact of premenstrual syndrome on quality of life. Pak J Med Sci.

[10] Akaberian S, Bahreini M, Afrasiabi S, Motamed N, Hajiloo M (2013). The Relationship between Premenstrual Syndrome and Personality Types among Female Students of Bushehr Universities, Iran. Iran J Obstet Gynecol Infertil.

[11] Delara M, Borzuei H, Montazeri A (2013). Premenstrual disorders: prevalence and associated factors in a sample of Iranian adolescents. Iran Red Crescent Med J.

[12] Cohn LD, Becker BJ (2003). How meta-analysis increases statistical power. Psychol Methods.

[13] Bazarganipour F, Taghavi SA, Montazeri A, Ahmadi F, Chaman R, Khosravi A (2015). The impact of polycystic ovary syndrome on the health-related quality of life: A systematic review and meta-analysis. Iran J Reprod Med.

[14] Vakilian K, Ranjbaran M, Khorsandi M, Sharafkhani N, Khodadost M (2015). Prevalence of preterm labor in Iran: A systematic review and meta-analysis. Int J Reprod BioMed.

[15] Von Elm E, Altman DG, Egger M, Pocock SJ, Gøtzsche PC, Vandenbroucke JP (2014). The Strengthening the Reporting of Observational Studies in Epidemiology (STROBE) Statement: guidelines for reporting observational studies. Int J Surg.

[16] Higgins JP, Thompson SG, Deeks JJ, Altman DG (2003). Measuring inconsistency in meta-analyses. BMJ.

[17] Rostami Dovom M, Ramezani Tehrani F, Farahmand M, Hashemi S, Rezaee N, Azizi F (2014). [Prevalence of menstrual disorders and its related factors in 18-45 year-old Iranian women in four selected provinces]. Hakim Res J.

[18] Bakhshandehnosrat S, Salehi M, Mobasheri E, Asghari Z, Mohammadkhani M (2014). [Prevalence of clinical manifestations of pre-menstrual syndrome and pre-menstrual dysphoric disorder among medical students in Gorgan, Iran (2010)]. J Gorgan Uni Med Sci.

[19] Shahghaibi S, Darvishi N, Yousefinejad V, Moghbel N, Shahsavari S (2009). [Investigation of the incidence rate of menstrual disorders in 17 and 18 year old high school female students in Sanandaj city in 2005]. Sci J Kurdistan Uni Med Sci.

[20] Mahmoodi Z, Shahpoorian F, Bastani F, Parsay S, Hoseini F, Amini L (2010). The prevalence and severity of premenstrual syndrome (pms) and its' associated signs and symptoms among college students. Aust J Basic Appl Sci.

[21] Basirat Z, Haji Ahmadi M (2006). Evaluation of Dysmenorrhea and Premenstrual Syndrome in High School Girls in Babol. Iran J Obstet Gynecol Infertil.

[22] Talaei A, Fayyazi Bordbar MR, Nasiraei A, Pahlavani M, Dadgar S, Samari AA (2009). Epidemiology of Premenstrual Syndrome (PMS) in Students of Mashhad University of Medical Sciences. Iran J Obstet Gynecol Infertil.

[23] Ghafari F, Porgaznien T (2006). The Relationship of Severity Premenstrual Syndrome With Anger in Adolescent Girls. Iran J Obstet Gynecol Infertil.

[24] Bakhshani N, Hasanzadeh Z, Raghibi M (2012). Prevalence of premenstrual symptoms and premenstrual dysphoric disorder among adolescents students of Zahedan. Zahedan J Res Med Sci.

[25] Alavi A, Salahimoghadam AR, Alimalayeri N, Ramezanpour A (2007). Prevalence of clinical manifestations of premenstrual syndrome and. Hormozgan Med J.

[26] Amiri Farahani L, Farokhi F, Abbasi A (2014). [Prevalence, Severity, and Clinical Manifestations of Premenstrual Syndrome among the Students Residing in the Dormitories of Arak University of Medical Sciences, Iran]. Qom Univ Med Sci J.

[27] Azarnive MS, Tavakoli-Khormizi SA (2016). Relationship between levels of physical activity with pre menstrual syndrome among female university students. Sci J Hamadan Nurs Midwif Facult.

[28] Sadr SS, Samimi Ardestani SM, Razjouyan K, Daneshvari M, Zahed G (2014). Premenstrual Syndrome and Comorbid Depression Among Medical Students in the Internship Stage: A Descriptive Study. Iran J Psychiatry Behav Sci.

[29] Hariri FZ, Moghaddam-Banaem L, Siah Bazi S, Saki Malehi A, Montazeri A (2013). The Iranian version of the Premenstrual Symptoms Screening Tool (PSST): a validation study. Arch Women's Ment Health.

[30] Farrokh-Eslamlou H, Oshnouei S, Heshmatian B, Akbari E (2015). Premenstrual syndrome and quality of life in Iranian medical students. Sex Reprod Healthc.

[31] Bakhshani NM, Mousavi MN, Khodabandeh G (2009). Prevalence and severity of premenstrual symptoms among Iranian female university students. J Pak Med Assoc.

[32] Masoumi SZ, Alamoti MK, Shobeiri F, Roshanaei G, Mohaghahi H (2016). Evaluating the prevalence of premenstrual syndrome among female undergraduate students of School of Nursing and Midwifery, Hamadan university of medical sciences in Iran. Res J Pharm Biol Chem Sci.

[33] Barkhordari F, Taavoni S, Haghani H (2013). Students' premenstrual symptoms severity in dorms of Tehran University of Medical Sciences. Giorn It Ost Gin.

[34] Zandi G, Onsory K, Helalat SH, Mirzaee S, Agha Alikhani E, Sadeghi Harsini M (2013). Prevalence of premenstrual syndrome and premenstrual dysphonic disorder among thestudents of Islamic Azad University of Parand. New Cell Mol Biotech J.

[35] Nourjah P (2008). Premenstrual syndrome among teacher training university students in Iran. J Obstet Gynecol India.

[36] Cheng SH, Shih CC, Yang YK, Chen KT, Chang YH, Yang YC (2013). Factors associated with premenstrual syndrome-a survey of new female university students. Kaohsiung J Med Sci.

[37] Potter J, Bouyer J, Trussell J, Moreau C (2009). Premenstrual syndrome prevalence and fluctuation over time: results from a French population-based survey. J Women's Health.

[38] Raval CM, Panchal BN, Tiwari DS, Vala AU, Bhatt RB (2016). Prevalence of premenstrual syndrome and premenstrual dysphoric disorder among college students of Bhavnagar, Gujarat. Indian J Psychiat.

[39] Derman O, Kanbur NÖ, Tokur TE, Kutluk T (2004). Premenstrual syndrome and associated symptoms in adolescent girls. Eur J Obstet Gynecol Reprod Biol.

[40] Takeda T, Koga S, Yaegashi N (2010). Prevalence of premenstrual syndrome and premenstrual dysphoric disorder in Japanese high school students. ArchWomen's Ment Health.

[41] Dueñas JL, Lete I, Bermejo R, Arbat A, Pérez-Campos E, Martínez-Salmeán J (2011). Prevalence of premenstrual syndrome and premenstrual dysphoric disorder in a representative cohort of Spanish women of fertile age. Eur J Obstet Gynecol Reprod Biol.

[42] Antai A, Udezi A, Ekanem E, Okon U, Umoiyoho A (2004). Premenstrual syndrome: Prevalence in students of the University of Calabar, Nigeria. Afr J Biomed Res.

[43] Tolossa FW, Bekele ML (2014). Prevalence, impacts and medical managements of premenstrual syndrome among female students: cross-sectional study in College of Health Sciences, Mekelle University, Mekelle, northern Ethiopia. BMC Womens Health.

[44] Bianco V, Cestari AM, Casati D, Cipriani S, Radici G, Valente I (2014). Premenstrual syndrome and beyond: lifestyle, nutrition, and personal facts. Minerva Ginecol.

[45] Balaha MH, Amr MA, Saleh Al Moghannum M, Saab Al Muhaidab N (2010). The phenomenology of premenstrual syndrome in female medical students: a cross sectional study. Pan Afr Med J.

[46] Buddhabunyakan N, Kaewrudee S, Chongsomchai C, Soontrapa S, Somboonporn W, Sothornwit J (2017). Premenstrual syndrome (PMS) among high school students. Int J Women's Health.

